# The Relationship of Oxidation Sensitivity of Red Blood Cells and Carbonic Anhydrase Activity in Stored Human Blood: Effect of Certain Phenolic Compounds

**DOI:** 10.1155/2016/3057384

**Published:** 2016-06-20

**Authors:** Zübeyir Huyut, Mehmet Ramazan Şekeroğlu, Ragıp Balahoroğlu, Tahsin Karakoyun, Erdem Çokluk

**Affiliations:** Department of Biochemistry, Medical Faculty, Yüzüncü Yıl University, 65080 Van, Turkey

## Abstract

It has been reported that many modifications occur with the increase of oxidative stress during storage in erythrocytes. In order to delay these negative changes, we evaluated whether the addition of substances likely to protect antioxidant capacity in stored blood would be useful. Therefore, we investigated the effects of resveratrol, tannic acid, and caffeic acid in lipid peroxidation and antioxidant capacity of erythrocytes in stored blood. Donated blood was taken into four CPD containing blood bags. One bag was used as the control, and the others were supplemented with caffeic acid (30 *μ*g/mL), resveratrol (30 *μ*g/mL), and tannic acid (15 *μ*g/mL), respectively. Erythrocyte lipid peroxidation, sensitivity to oxidation, glutathione levels and carbonic anhydrase, glutathione peroxidase, and catalase activities were measured on days 0, 7, 14, 21, and 28. In the control group, erythrocyte malondialdehyde levels and sensitivity to oxidation were increased whereas glutathione, glutathione peroxidase, and catalase levels were decreased (*p* < 0.05). Resveratrol and caffeic acid prevented malondialdehyde accumulation and preserved glutathione, glutathione peroxidase, and catalase activities in erythrocytes. We demonstrated that resveratrol, caffeic acid, and tannic acid in stored blood could decrease the sensitivity to oxidation of erythrocytes in vitro but did not exhibit such effects on CA activity.

## 1. Introduction

The treatment of many diseases (e.g., acute blood loss, injury, and anemia) by blood transfusion can be successful [1]. The packed red blood cells (RBCs) for transfusion can be stored for approximately up to 42 days at 2–6°C [2, 3]. It has been reported that some changes occur during the course of storage [4]. During storage, progressive morphological and biochemical changes occur which are often related to the reduction of ATP, 2,3-biphosphoglycerate, and NADH in red blood cells (RBCs) [5, 6]. These changes are referred to as the “storage lesions” [5]. There is also strong evidence that oxidative stress plays a major role in the storage lesions [7]. In a previous study, we showed that storage time can increase sensitivity to oxidation and oxidative stress in RBCs [7]. In addition, we also revealed a decrease in levels of reduced glutathione (GSH) and activity of antioxidant protective enzymes such as glutathione peroxidase (GSH-Px), catalase, and superoxide dismutase (SOD) [7]. These negative changes shorten the shelf lives of stored blood [3]. When stored blood cannot be used in time, it is discarded; therefore, the loss of budget, labour, and time came into question.

The carbonic anhydrase enzyme (CA, EC.4.2.1.1) including the Zn(II) ion catalyses the reversible hydration of carbon dioxide to bicarbonate and protons. This enzyme has 16 different isoenzymes that are currently known in human [8–10]. CA I and CA II are the major isozymes that are present at high concentrations in the cytosol in RBC [11, 12]. CA participates in the maintenance of pH homeostasis or erythrocytes by catalysing the reversible hydration of carbon dioxide.

Several preservatives are added to stored blood, such as antioxidants to retard these negative changes [7]. The optimum protective solution used to protect erythrocytes provides optimal oxygen release and maximum viability of RBC [1]. Citrate-phosphate-dextrose (CPD), acid-citrate-dextrose (ACD), citrate-phosphate-dextrose-adenine (CPDA-1), and sorbitol-adenine-glucose-mannitol (SAGM) are the most common combination that is added to the stored blood [3, 13]. However, irrespective of the additive used in preparing the packed cell units, studies also indicate that blood kept under proper circumstances is subject to increased lipid peroxidation and decreased antioxidant capacity, due to the storage time [14].

Antioxidants are molecules which are exogenous or endogenous. These molecules neutralize the oxidative damage caused by oxidants by their own intra- and extracellular defence mechanisms. The extracellular defence mechanisms include various molecules, such as albumin, bilirubin, transferrin, ceruloplasmin, uric acid, ascorbate, and *α*-tocopherol. Intracellular free radical-scavenging enzymes provide the primary antioxidant defence mechanism. These enzymes are superoxide dismutase (SOD), glutathione-S-transferase and glutathione peroxidase (GSH-Px), glutathione reductase, catalase, and cytochrome oxidase [14, 15].

The antioxidant properties of phenolic compounds are mainly due to their strong reduction potential and ability to give electrons and protons, remove singlet oxygen, and chelate the metal ions [16]. In our study, resveratrol, tannic acid, and caffeic acid are antioxidants from natural origins [16–18]. Resveratrol is a natural compound acting on a series of intracellular mediators. Due to its beneficial effects on the human body, it has become a focus of interest all over the world [16]. Tannic acid is a naturally occurring polyphenol content of phenolic compounds found in some plants and fruits [18]. Some nutrients include tannic acid such as wine, beer, coffee, black tea, pears, bananas, black-eyed peas, lentils, and chocolate [19, 20]. It is reported that caffeic acid (3,4-dihydroxycinnamic acid), another substance of our study, acts in a protective role in the low density lipoproteins to *α*-tocopherol [21]. In previous studies, it was shown that resveratrol, caffeic acid, and tannic acid had better antioxidant capacity at 30, 30, and 15 *μ*g/mL concentrations in vitro, respectively [16–18].

In our study, we investigated the effects of resveratrol, caffeic acid, and tannic acid on lipid peroxidation, sensitivity to oxidation, and activity of carbonic anhydrase (CA) that protect pH balance in stored blood, in the red blood cells of the stored blood. In addition, we determined the effects of these molecules on catalase, GSH-Px, and reduced glutathione (GSH) levels which provide the main intracellular antioxidant defence system in RBC.

## 2. Material and Methods

The study protocol was performed in accordance with the Helsinki Declaration as revised in 2000. The study was also approved by the Ethics Committee of the Yüzüncü Yıl University, and all patients provided their consent. Blood samples were collected into quaternary paediatric pouch systems (Kansuk®, Turkey) containing citrate-phosphate-dextrose (CPD) from 10 healthy male volunteers between 20 and 30 years of age. Blood pouches contained 500 mL blood and 70 mL CPD solution. Each 500 mL blood sample was separated into 4 paediatric pouches connected to the main pouch. The first group was used as the control group with no substance added to the blood. A total of 30 *μ*g/mL caffeic acid and 30 *μ*g/mL resveratrol and 15 *μ*g/mL tannic acid were added to the second, third, and fourth group, respectively. Next, 10 mL blood samples were taken in each group on day 0 and on the 7th, 14th, 21st, and 28th days, respectively, and centrifuged at 2500 g, and the plasma was separated. Equal volumes of isotonic solution and RBC were added to the tubes and centrifuged at 2500 g. The supernatant was aspirated and this washing procedure was repeated three times. MDA levels and the susceptibility to oxidation were quantified from RBC suspension on the same days. The RBC packages were stored at −20°C until catalase and GSH-Px and GSH levels were measured.

### 2.1. Haemoglobin Measurement of Erythrocyte Packages

Haemoglobin was measured by the method of Fairbanks and Klee which utilizes cyanomethemoglobin [22]. For this purpose, 5 mL Drabkin solution and 20 *μ*L haemolysate were added to tubes and incubated for 10 minutes at room temperature. The absorbance was measured colorimetrically at 540 nm in spectrophotometer (Shimadzu UV mini 1240), and haemoglobin concentration was represented in terms of g/dL.

### 2.2. Measurement of MDA

MDA levels of RBC suspensions were analyzed by high-performance liquid chromatography (HPLC), as described by Khoschsorur et al. [23]. The mixture (50 *μ*L erythrocytes suspension, 0.44 M and 750 *μ*L H_3_PO_4_, and 42 mM and 250 *μ*L TBA) was incubated for the formation of the MDA-thiobarbituric acid (TBA) coloured complex. MDA-TBA complex was detected fluorometrically at an excitation wavelength of 527 nm and at an emission wavelength of 551 nm. We used Agilent 1200 series HPLC systems with an autosampler, a gradient pump, a column frame, a fluorescence detector (FLD) (Agilent Technologies, Waldbronn, Germany), and RP-C18 analytical column (150 × 4.6 mm, 5 *μ*m particle size, ACE, Aberdeen, Scotland). Phosphate buffer (pH: 6.8, 50 mM) and methanol (v/v: 4/6) were used as mobile phase by adding them to each other. The MDA-TBA adduct peak was calibrated using a 1,1,3,3-tetraethoxypropane (Sigma-Aldrich, USA) standard solution, and the MDA levels were expressed in nmol/gHb.

### 2.3. Susceptibility to Oxidation of Erythrocyte

The method of Stocks et al. was used to measure the sensitivity of RBC to in vitro oxidation induced by H_2_O_2_ (in concentration 0.03% in buffer saline solution) in RBC suspension [24]. In this method, the RBC suspension (5 mL) was prepared using azide buffer and RBC. Next, 5.5 mL of H_2_O_2_ solution was added to the same cell suspension and it was incubated at 37°C for 2 h. The MDA level was measured again with TBA as mentioned above (Section 2.2.) (2 h). Haemoglobin concentration of the erythrocyte suspension was also measured as mentioned above (Section 2.1.) and MDA levels were expressed as nmol/gHb.

### 2.4. Measurement of GSH-Px Activity

Erythrocyte GSH-Px activity was determined using the method described by Paglia and Valentin [25]. In the presence of glutathione reductase and NADPH, the oxidized glutathione is immediately converted to the reduced form with concomitant oxidation of NADPH to NADP^+^. The decrease in absorbance at 340 nm in spectrophotometer (Shimadzu UV mini 1240) was measured every 30 seconds, six times, for 3 minutes. The (Δ*A*) absorbance change was calculated and the obtained results were expressed as IU/gHb.

### 2.5. Measurement of Catalase Activity

Erythrocyte catalase activity was measured according to the colorimetric method of Góth [26]. The 0.2 mL sample was incubated with 1 mL H_2_O_2_ (65 *μ*mol/L in phosphate buffer, pH 7.4) for one minute, and the reaction was stopped by adding 1 mL ammonium molybdate solution (32.4 mmol/L). The absorbance of the yellow complex formed by molybdate and H_2_O_2_ was measured at 405 nm spectrophotometrically in spectrophotometer (Shimadzu UV mini 1240). The obtained results are expressed as kIU/gHb.

### 2.6. Measurement of Reduced GSH

RBC glutathione level was measured according to the spectrophotometric method of Fairbanks and Klee [27]. Virtually, all of the nonprotein sulfhydryl groups of erythrocytes are in the form of reduced GSH. 5,5′-Dithiobis(2-nitrobenzoic acid) (DTNB) is a disulphide chromogen that is readily reduced by sulfhydryl compounds to an intensely yellow compound. The absorbance of the reduced chromogen is measured at 412 nm in spectrophotometer (Shimadzu UV mini 1240) and is directly proportional to the GSH concentration. Although the DTNB assay measures all free sulfhydryl groups in protein and nonprotein, it is considered that the level of free sulfhydryl groups reflects the level of GSH in RBC because the GSH level in erythrocytes is notably high. The obtained results are expressed as mg/gHb.

### 2.7. Measurement of Carbonic Anhydrase Activity

Total CA enzyme activity in RBC was measured by using the method of Rickly et al. that is modified by Wilbur and Anderson [28, 29]. The enzymatic reaction in a total volume of 1.7 mL contained 1 mL veronal buffer (pH 8.2), 0.1 mL bromine thymol blue (0.04%), 0.55 mL pure water, and 0.05 mL enzyme solution. A reference measurement was obtained by preparing the same cuvette without enzyme solution. Carbonic anhydrase activity (CO_2_-hydratase activity) was assayed by following the hydration of CO_2_. CO_2_-hydratase activity as an enzyme unit (EU) was calculated by using the equation *t*_0_ − *t*_*c*_/*t*_*c*_, where *t*_0_ and *t*_*c*_ are the times for pH change of the nonenzymatic and the enzymatic reactions, respectively [30].

### 2.8. Statistical Analysis

Descriptive statistics for continuous variables according to the obtained result were expressed as the mean ± standard deviation. Variance analysis (two-way ANOVA) was used to compare the groups and Duncan's multiple comparison was used to compare intergroups. *p* values less than 0.05 were considered to be significant. SPSS statistics package program (SPSS Inc., IL, USA, version 15) was used to perform statistical analysis.

## 3. Results and Discussion

### 3.1. Lipid Peroxidation and Susceptibility to Oxidation in Erythrocytes Packages

A major contributing factor to the decreasing lifespan of the stored erythrocytes could be a decrease in the antioxidant defence system or an increase in oxidative stress. Numerous studies have shown that, after transfusion, RBCs substantially lose their viability (approximately 30%) due to storage time [3, 7]. Erythrocytes are rich in nonconjugated polyunsaturated fatty acids with reactive methylene groups that are susceptible to hydrogen atom abstraction. The oxidation of these fatty acids by free radicals increases MDA levels, which are the end product of lipid peroxidation [31, 32]. MDA is the end product of lipid peroxidation and formed due to the effect of free radicals that modify lipid and lipid derivative molecules. Oxidant molecules, such as superoxide anion (O_2_^•−^), H_2_O_2_, and the hydroxyl radical (HO^•−^), may occur during the storage period by the influence of some factors. They may cause damage to the protein and lipid structures of erythrocytes [32].

It has also been reported that the functions of membrane phospholipids and proteins are negatively affected by MDA [7, 32]. A decrease in antioxidant capacity and an increase in lipid peroxidation of the erythrocytes in the stored blood during the storage period have been reported in various studies [32, 33]. Sekeroğlu et al. [7] reported that MDA and GSH levels and GSH-Px, SOD, and catalase activities were decreased by lipid peroxidation time-dependently in the RBC of the stored blood. Additionally, Aslan et al. [14] showed that MDA levels in stored blood decreased. The additive solutions used in preparing packed cell units were not initially designed to reduce oxidative stress per se; most were designed to try to maintain 2,3-DPG levels, pH, and so forth. Some of the components do however have antioxidant properties. There have been numerous studies to increase the storage time of the blood by adding preservative substances to reduce oxidative stress or increase the antioxidant capacity of the RBC [3, 7]. In the literature, there has been no other study that has examined the protective effect of resveratrol, caffeic acid, and tannic acid on oxidative stress and the antioxidant capacity of RBC in stored blood.

In our study, comparisons in all groups compared to baseline MDA levels of RBC were higher from the 7th day (*p* < 0.05). However, MDA levels of caffeic acid group on the 14th, 21st, and 28th days were lower than MDA levels of other groups on the same days (Table 1, *p* < 0.05). In addition, we also found that erythrocyte MDA levels increased with storage time in the stored blood. This finding is consistent with previous studies and supports the view that oxidative damage of the erythrocytes membrane during storage renders them more susceptible to further oxidative stress.

We also tested the sensitivity to in vitro oxidation of stored RBC induced by hydrogen peroxide. The effects of caffeic acid, tannic acid, and resveratrol on the sensitivity to oxidation of RBC 2 hours after adding the solution of H_2_O_2_ are shown in Table 2. Intragroup comparisons showed that the sensitivity to oxidation of RBC increased from the 7th day compared to the baseline (*p* < 0.05). However, the sensitivity to oxidation of RBC in the caffeic acid and resveratrol groups on the 21st and 28th days was lower than in the control group (*p* < 0.05). In addition, MDA levels of resveratrol group on the 21st and 28th days were lower than MDA levels of the control, tannic acid, and caffeic acid groups (Table 2, *p* < 0.05). These results showed that the susceptibility to oxidation of RBC is protected with the addition of caffeic acid and resveratrol. This property of resveratrol, caffeic acid, and tannic acid is consistent with the findings of previous studies in vitro [16–18].

### 3.2. Changes of Some Antioxidant Enzyme and Protein Values in Erythrocyte Packages

Dumaswala et al. [33] showed that membrane lipids and proteins were damaged due to the reduction of RBC antioxidants such as GSH and GSH-Px in stored blood. These researchers also showed accumulation of MDA, band 3 protein aggregation, and carboxylation of band 4.1 protein in stored blood which could adversely affect erythrocyte survival.

One of the most important reasons for the increase in oxidative stress in stored blood is the reduction in the antioxidant capacity. The major antioxidant systems in human erythrocytes are glutathione, SOD, GSH-Px, and catalase [7]. It has been reported that GSH, GSH-Px, and catalase act by removing H_2_O_2_ in RBC [34]. Gaetani et al. [35] showed that GSH-Px and catalase have equal active roles in the detoxification of H_2_O_2_ in human erythrocytes. It was reported that catalase and NADPH protect RBC against acute and high exogenous levels of H_2_O_2_, while GSH protects against the continuously produced low level of endogenous H_2_O_2_ [36]. Dumaswala et al. [37] showed that increasing the glutathione concentration in the stored blood protected the erythrocytes against free radical damage. Numerous studies have shown that antioxidant enzyme activity and antioxidant capacity decrease in stored blood in a time-dependent manner [7, 14]. Ziouzenkova et al. [38] showed that free haemoglobin level increased during blood storage and caused oxidative stress. Sekeroğlu et al. [7] showed that erythrocyte GSH, GSH-Px, and catalase levels were decreased during blood storage time. In this study, erythrocyte catalase activity was increased in the caffeic acid and resveratrol groups, whereas it was preserved in the tannic acid group on the 7th, 14th, 21st, and 28th days (*p* < 0.05). RBC catalase activity was decreased in the control group on the 28th day (*p* < 0.05). RBC catalase activity of the caffeic acid and resveratrol groups was higher than of the control group on the 7th, 14th, 21st, and 28th days and of the tannic acid group on the 14th, 21st, and 28th days. In addition, catalase activity of the caffeic acid group was higher than of the other groups on the 21st day (Table 3, *p* < 0.05).

In intragroups comparisons, GSH levels of RBC decreased on the 21st and 28th days in the control group and were preserved in the tannic acid group. On the contrary, the levels increased in the caffeic acid group on the 21st and 28th days and on the 7th, 14th, 21st, and 28th days in the resveratrol group, respectively (*p* < 0.05). GSH levels were higher in the resveratrol, caffeic acid, and tannic acid groups than in the control group on the 21st and 28th days (Table 4, *p* < 0.05). In addition, GSH levels were higher in the caffeic acid group than in the control and tannic acid groups on the 28th day (*p* < 0.05).

There was a similar situation in GSH-Px activity. In intragroups comparisons, erythrocyte GSH-Px activity was increased on the 7th, 14th, 21st, and 28th days in control group and on the 28th day in tannic acid group. In the caffeic acid and resveratrol groups, the activity of GSH-Px was increased on the 7th and 14th days and then decreased back to the baseline levels (Table 5, *p* < 0.05). In addition, the comparison between groups showed that the GSH-Px levels of resveratrol, caffeic acid, and tannic acid groups were higher than of the control group on the 7th, 14th, 21st, and 28th days (*p* < 0.05).

The levels of antioxidant enzyme and protein such as GSH, GSH-Px, and catalase were significantly increased at the beginning of the storage period and throughout the storage time, which was consistent with previous studies [39]. However, we observed that the addition of resveratrol, caffeic acid, and tannic acid protects the levels of GSH, GSH-Px, and catalase in stored blood and activates them during the first two weeks of storage time. We did not find any previous study in the literature concerning how phenolic compounds affect the erythrocyte GSH, GSH-Px, and catalase levels in in vitro human or animal models. But it was shown that tannic acid and resveratrol decreased lipid peroxidation and increased SOD, GSH, GSH-Px, and catalase activity in nickel-, lead-, and alcohol-induced oxidative stress, in tissue of kidney and liver as well as in vivo [40–42]. This situation may be caused by free radical-scavenging properties of phenolic compounds.

Decreasing of catalase and GSH levels is caused by increasing formation of H_2_O_2_ or free radicals time-dependently. The addition of resveratrol, caffeic acid, and tannic acid may be responsible for the increased activities of GSH-Px and catalase in the first two weeks of this study because of their properties of scavenging the free radicals or activating the catalase. Moreover, the molecular properties of phenolic compounds may lead to an increase in GSH levels. Resveratrol, caffeic acid, and tannic acid may scavenge the free radicals which occur in the stored blood. For this reason, resveratrol, caffeic acid, and tannic acid may become associated with the conversion of oxidized glutathione (GSSG) to the reduced form.

### 3.3. Changes of Total Carbonic Anhydrase Enzyme Activities in Erythrocytes Packages

CA enzyme catalyses the hydration (H^+^) of carbon dioxide (CO_2_) to bicarbonate (HCO_3_^−^) and the corresponding dehydration of bicarbonate in acidic medium with regeneration of CO_2_, in a two-step reaction [43]. It is clearly an important role in ensuring proper pH environment to sustain antioxidant capacity and the life of the erythrocytes. Therefore, the preservation of the CA activity is very important for pH balance in the stored blood or erythrocytes. There is no other study in the literature that has determined time-dependent change in CA activity of the RBC in stored blood. In our study, erythrocyte total CA activity in the control group decreased time-dependently, but it is of no statistical significance (Table 6). Erythrocyte CA activities of the resveratrol, tannic acid, and caffeic acid groups were significantly higher on the 14th day compared to the respective baseline levels (*p* < 0.05, Table 6). However, the activity of CA decreased back to the baseline over time. This increase only continued past the 21st day in the caffeic acid group (*p* < 0.05).

pH value is another parameter associated with CA activity. If the pH changes, the erythrocyte membrane integrity is impaired and an increase in erythrocyte oxidation is seen [1]. In our study, baseline (time 0) pH values were similar in all groups (in the range of 7.037 ± 0.073 and 7.08 ± 0.114) and there was no statistical difference between the groups. The pH levels gradually dropped as time progresses (*p* < 0.05, control group: 6.583 ± 0.055). But pH values of the 28th day in groups of resveratrol and caffeic acid were higher compared to the control group (*p* < 0.05, 6.590 ± 0.051, and 6.627 ± 0.073, resp.).

These results demonstrated that pH levels decreased time-dependently, but the resveratrol and caffeic acid partially protected pH changes. This situation may be caused from partially protective effect of resveratrol and caffeic acid on the CA activity.

## 4. Conclusions

This study demonstrated that lipid peroxidation and sensitivity to oxidation increased and the antioxidant capacity decreased in RBC of the stored blood time-dependently. However, the caffeic acid, resveratrol, and tannic acid partially prevented the lipid peroxidation in RBC. Also, in RBC, they decreased the sensitivity to oxidation of RBC by protecting antioxidant enzyme activities such as GSH-Px, catalase, and GSH. In addition, caffeic acid and resveratrol partially prevented the CA activity and pH change time-dependently. This situation explains why resveratrol and caffeic acid can better prevent lipid peroxidation in RBC. Furthermore, because the phenolic compounds are able to remove the free radicals forming in the stored blood time-dependently, resveratrol, tannic acid, and caffeic acid may have increased the activity of GSH, GSH-Px, and catalase. But, nevertheless, in vitro kinetic studies are needed to explain the molecular effects of resveratrol, caffeic acid, and tannic acid on the increase of erythrocyte GSH-Px, GSH, and catalase activities.

Based on these results, resveratrol, caffeic acid, and tannic acid may provide a positive contribution to the extension of the shelf life of stored blood and the conservation of the antioxidant capacity in RBC. But their influence on factors such as osmotic fragility, total antioxidant capacity, total oxidative stress, and protein carbonyl was investigated. Additionally, to minimise their possible side effects such as acidosis, they must be used carefully and their dosages must be adjusted correctly.

## Figures and Tables

**Table 1:** The inhibition effects of caffeic acid, resveratrol, and tannic acid on erythrocyte MDA levels in stored whole blood time-dependently.

MDA (nmol/gHb)				
	Control	Caffeic acid	Resveratrol	Tannic acid
Baseline (time 0)	22.33 ± 6.37	21.28 ± 5.13	21.97 ± 8.08	21.29 ± 6.49
7th day	35.70 ± 2.89^҂^	29.64 ± 1.67^*҂*,*∗*^	31.09 ± 4.79^*҂*,*∗*^	31.43 ± 3.01^*҂*,*∗*^
14th day	37.36 ± 5.28^҂^	32.20 ± 2.76^*҂*,*∗∗*^	36.91 ± 1.98^*҂*^	37.68 ± 5.20^҂^
21st day	39.94 ± 6.95^҂^	33.86 ± 2.91^*҂*,*∗∗*^	40.26 ± 4.92^*҂*^	44.41 ± 6.02^*҂*,*∗*^
28th day	46.04 ± 9.87^҂^	38.51 ± 1.53^*҂*,*∗∗*^	45.87 ± 5.54^*҂*^	46.13 ± 6.76^҂^

Significant differences (*p* < 0.05) between the control and treated groups are indicated by *∗*.

Significant differences (*p* < 0.05) between the control and resveratrol and tannic acid are indicated by *∗∗*.

Significant differences (*p* < 0.05) in each group from time 0 (baseline) are given by ҂.

**Table 2:** The inhibition effects of caffeic acid, resveratrol, and tannic acid on erythrocyte MDA levels after the two-hour incubation with the solution of H_2_O_2_ (sensitivity to oxidation of RBC).

2-hour MDA (nmol/gHb)				
	Control	Caffeic acid	Resveratrol	Tannic acid
Baseline	101.96 ± 9.14	103.56 ± 14.40	107.96 ± 9.14	108.81 ± 11.38
7th day	274.75 ± 21.93^҂^	309.19 ± 28.20^*҂*,*∗*^	221.28 ± 15.69^҂^	267.93 ± 24.71^*҂*,*∗*^
14th day	397.54 ± 23.38^҂^	390.10 ± 32.71^҂^	298.09 ± 29.89^҂^	421.70 ± 27.15^*҂*,*∗*,*∗∗*^
21st day	437.84 ± 19.96^҂^	407.85 ± 38.35^*҂*,*∗*^	314.02 ± 18.41^*҂*,*∗*,*∗∗*^	433.26 ± 18.82^҂^
28th day	499.69 ± 12.60^҂^	411.51 ± 20.15^*҂*,*∗*^	338.85 ± 15.83^*҂*,*∗*,*∗∗*^	441.73 ± 20.17^҂^

Significant differences (*p* < 0.05) between the control and treated groups are indicated by *∗*.

Significant differences (*p* < 0.05) between the control and caffeic acid and tannic acid are indicated by *∗∗*.

Significant differences (*p* < 0.05) in each group from time 0 (baseline) are given by ҂.

**Table 3:** The protective effects of caffeic acid, resveratrol, and tannic acid on catalase activity of RBC in stored whole blood time-dependently.

Catalase (kU/gHb)				
	Control	Caffeic acid	Resveratrol	Tannic acid
Baseline	17.47 ± 2.12	17.96 ± 4.49	18.02 ± 6.76	19.12 ± 1.53
7th day	16.52 ± 3.04	30.12 ± 9.97^*҂*,*∗*^	38.64 ± 11.32^*҂*,*∗*^	19.52 ± 3.36
14th day	14.45 ± 1.56	40.94 ± 5.33^*҂*,*∗*^	40.96 ± 5.97^*҂*,*∗*^	20.26 ± 2.99^*∗*^
21st day	12.70 ± 1.51	56.22 ± 8.42^*҂*,*∗*,*∗∗*^	50.15 ± 13.57^*҂*,*∗*^	20.89 ± 2.08^*∗*^
28th day	9.16 ± 1.52^҂^	48.84 ± 11.62^*҂*,*∗*^	47.57 ± 7.32^*҂*,*∗*^	20.47 ± 1.73^*∗*^

Significant differences (*p* < 0.05) between the control and treated groups are indicated by *∗*.

Significant differences (*p* < 0.05) between the control and resveratrol and tannic acid are indicated by *∗∗*.

Significant differences (*p* < 0.05) in each group from time 0 (baseline) are given by *҂*.

**Table 4:** The protective effects of caffeic acid, resveratrol, and tannic acid on the GSH levels of RBC in stored whole blood time-dependently.

GSH (mg/gHb)				
	Control	Caffeic acid	Resveratrol	Tannic acid
Baseline	10.72 ± 0.68	9.80 ± 2.44	9.94 ± 0.69	10.34 ± 1.59
7th day	10.51 ± 1.15	10.96 ± 1.52	12.86 ± 1.20^҂^	10.27 ± 1.20
14th day	10.36 ± 0.63	10.61 ± 0.72	14.09 ± 1.76^*҂*,*∗*^	11.00 ± 1.75
21st day	8.53 ± 1.94^҂^	12.52 ± 2.29^*҂*,*∗*^	12.46 ± 0.91^*҂*,*∗*^	10.71 ± 1.44^*∗*^
28th day	7.96 ± 0.59^҂^	12.32 ± 1.54^*҂*,*∗*,*∗∗*^	12.67 ± 0.52^*҂*,*∗*^	10.57 ± 1.31^*∗*^

Significant differences (*p* < 0.05) between the control and treated groups are indicated by *∗*.

Significant differences (*p* < 0.05) between the control and tannic acid are indicated by *∗∗*.

Significant differences (*p* < 0.05) in each group from time 0 (baseline) are given by *҂*.

**Table 5:** The protective effects of caffeic acid, resveratrol, and tannic acid on GSH-Px activity of RBC in stored whole blood time-dependently.

GSH-Px (IU/gHb)				
	Control	Caffeic acid	Resveratrol	Tannic acid
Baseline	50.35 ± 2.38	48.92 ± 3.73	49.14 ± 2.81	50.29 ± 4.24
7th day	45.47 ± 2.89^҂^	50.93 ± 3.36^*∗*^	52.09 ± 2.25^*҂*,*∗*^	50.82 ± 1.81^*∗*^
14th day	42.79 ± 2.59^҂^	53.07 ± 2.87^*҂*,*∗*^	53.43 ± 2.61^*҂*,*∗*^	51.38 ± 1.93^*∗*^
21st day	37.05 ± 2.04^҂^	51.20 ± 3.26^*∗*^	50.35 ± 2.32^*∗*^	49.76 ± 1.51^*∗*^
28th day	28.69 ± 3.91^҂^	46.65 ± 3.03^*∗*^	48.90 ± 2.38^*∗*^	45.30 ± 1.74^*҂*,*∗*^

Significant differences (*p* < 0.05) between the control and treated groups are indicated by *∗*.

Significant differences (*p* < 0.05) in each group from time 0 (baseline) are given by *҂*.

**Table 6:** The protective effects of caffeic acid, resveratrol, and tannic acid on carbonic anhydrase activity of RBC in stored whole blood time-dependently.

Carbonic anhydrase activity (IU/gHb)				
	Control	Caffeic acid	Resveratrol	Tannic acid
Baseline	37447.05 ± 6610.71	35459.50 ± 6875.23	33061.22 ± 3328.76	39592.14 ± 4641.80
7th day	35759.53 ± 5841.47	40489.28 ± 5857.81	40213.21 ± 7552.89^҂^	43124.78 ± 11072.50^*∗*^
14th day	34881.80 ± 6910.69	44055.82 ± 10547.32^*҂*,*∗*^	46072.18 ± 11850.02^*҂*,*∗*^	43289.98 ± 7619.90^*∗*^
21st day	34822.20 ± 2570.16	47601.52 ± 16160.80^*҂*,*∗*,*∗∗*^	36897.59 ± 4755.26	38277.01 ± 6653.30
28th day	33714.45 ± 4760.06	40182.18 ± 6503.96	36858.38 ± 5734.33	37319.28 ± 4357.00

Significant differences (*p* < 0.05) between the control and treated groups are indicated by *∗*.

Significant differences (*p* < 0.05) between the control and resveratrol and tannic acid are indicated by *∗∗*.

Significant differences (*p* < 0.05) in each group from time 0 (baseline) are given by *҂*.
